# Taking psychiatry to the public in the Third World: Potential and pitfalls

**DOI:** 10.4103/0019-5545.55933

**Published:** 2005

**Authors:** Nimesh G. Desai

The need for and correctness of knowledge reaching the larger proportion of the human population, ‘the masses’ or ‘the public’, is high in almost all disciplines but particularly so in science because it is meant to serve the purpose of improving human existence. The growth and expansion of science has made this task progressively more difficult due to the increasingly ‘technical’ nature of scientific concepts and language. Although the recognition for public education in science or carrying science to the public is reaching imperative levels, the strategies and mechanisms for doing so are becoming more complex.

The justification for disseminating knowledge to the public in the field of medical or health sciences is unarguable, since it has the potential to enhance participation in treatment and other health-related behaviour. The need for effective communication of scientific knowledge on illness and health is matched by the complex nature of the communication strategies required to disseminate the ‘technical’ nature of information and knowledge. These complexities need to be factored in to ensure that the correct scientific information becomes available to the public for better understanding and implementation.

Psychiatry, a medical discipline and yet with significant differences, has its advantages and disadvantages in reaching its message to the public. The larger field of mental health, of course, has its own dilemmas in public education, what with its issues of multidisciplinary identity and amorphous concepts. The debate whether psychiatry or mental health should be the focus of public education is a valid one in itself, but to examine the task of taking psychiatry to the public is a useful first step. Psychiatry, being less technology-based as compared to other medical disciplines, should have had an advantage in its conceptualizations and syntax, and yet this does not seem to have been the case. ‘Psychobabble’ or psychological ‘mumbo–jumbo’ does not enthuse the public or help in understanding the concepts. The fact that psychiatry deals with abnormal functioning of the psyche, using terms from psychology, which have different connotations in day-to-day parlance, makes the task of communicating these concepts more difficult. Moreover, the belief of the lay people in general, and of opinion-makers in particular, that they already know enough about the functioning of the human mind, if not also the reasons thereof, renders them not very receptive to the efforts by professionals. The self-proclaimed ‘expertise’ that many members of the public seem to have on issues of psychiatry does make the task of taking the concepts in psychiatry to the public difficult.

It has been argued that the distinction between writings in psychiatry and those in journalism were narrowed because ‘as with everything else, we can blame Freud who wrote directly for the public, and whose case histories can be consumed as fiction’.^1^ It must also be recognized that it was Freud who was most influential in taking the concepts to the public. Herein lies one inherent pitfall. The scientific concepts in psychiatry to be conveyed to the public will have to be communicated in a style and syntax that is simple and narrative, and yet avoid the risk of becoming too non-scientific or journalistic. This dilemma of maximizing the potential of reaching out to the larger public in a meaningful manner and the pitfall of compromising on the scientific content is reflected much too often in the efforts by professionals in the popular media. On the other hand, the considerable sensationalization of stories related to mentally ill persons or their actions by journalists is often seen as excessive by professionals. The impact of such patterns on suicidal behaviour has been controversial and, although the evidence is not substantive, the print media in the western world generally seem to have a self-imposed code of not highlighting the details of the method of suicide and of toning down excesses.^1^

It has recently been documented that the public in the UK seemed largely ignorant of the work of psychiatrists.^2^ Some argue that it is reticence on the part of psychiatrists that contributes to the lack of knowledge. An experiment in Norway showed that a coordinated press campaign reduced the duration of untreated psychosis in one of the counties from 118 weeks to 26 weeks.^1^,^3^ Effective collaboration between psychiatrists and media workers has been difficult because of the differences in their respective functioning and approaches. While media workers are narrative-based, creative, plain-speaking, anti-authority and guardians of the public interest with emphasis on the public's right to know; psychiatrists are evidence-based, scientific, contemplative, jargon-using guardians of the public, with the added role of being keepers of their patients' secrets.^1^ But are these differences impossible to transcend?

The proliferation of the media of mass communication from radio to television and the internet makes it difficult for psychiatrists to stay insulated. At the same time, the lure of appearing on the radio or television does not always match the substantiveness of the message intended to be conveyed. One producer has recommended daytime television as a suitable forum for mental health issues, while the counterview is that no group which has successfully fought discrimination has achieved equality by arousing pity, and so ‘television is the worst medium through which to consider exploring the complex biopsychosocial origins of mental illness and its treatment, and such programmes are [almost] all in the worst possible taste’.^1^,^3^ The large proportion of violence-related issues in the television news coverage of mental health issues has been cited to suggest that ‘television in general, and television news in particular, is a major reservoir of stigma’.^4^ The tendency of some psychiatrists to opine on every aspect of medical, social and political life or ‘demand hegemony over them’^5^ has been cautioned against in the western world and is a clear danger in Third World countries.^1^ The dilemma here obviously is of the potential of reaching out to a large number of people through the audiovisual media and the pitfall is the risk of being too eager to analyse or put a label on every aspect of human behaviour. It is also questionable whether the public perception of, or attitude towards, mentally ill persons is modified by the didactic approach adopted by television programmes or documentary films on mental illness. The pitfall of increasing the stigma by ill-conceived documentary approaches or merely losing sight of people's interest is significant, but needs to be countered by actively exploring the potential for narrative-based or simulated portrayals.

Barring a few exceptions, popular cinema has generally been known to depict mentally ill persons and psychiatrists in a negative light. The debate between the creativity of the artist, namely the director or the scriptwriter, versus their social responsibility, is delicately balanced. Ever so often, psychiatrists and their associations protest about the portrayal of a mentally ill person or a psychiatrist in a particular film, even when dispassionate understanding would suggest that the said portrayal was the reflection of the prevalent social perception of the phenomenon. The pitfall remains that over-protestation would jeopardize the opportunity of changing such perceptions and portrayals!

The role of effective services, especially if they are delivered in a user-friendly manner, in taking psychiatry to the public, has been not adequately recognized, despite the evidence about its usefulness,^6^,^7^ although not everyone in clinical psychiatry services is keen to share information with patients.^8^ It has been found in the UK that most patients would like their psychiatrists to consider their preferences and want to be involved in decisions about their care.^9^ There is no reason to believe that it would be any different in the Third World because it has been suggested that clinicians generally underestimate their patients’ desire for information.^10^

Most importantly, a systematic evaluation of awareness programmes by the Mental Health Awareness Action (MHAA) programme in the UK has found that the key active ingredient identified by all groups was the testimonies of service users. The statements of service users (consumers) about their experience of mental health problems and of their contact with a range of services had the greatest and most lasting impact on the target audiences in terms of reducing the mental health stigma.^11^

It would seem that besides other activities that we may involve ourselves in, one of the best, if not the surest, way of taking psychiatry to the public is to carry out our clinical work competently and sensitively, as well as relate with the experiences of consumers.
